# Betacyanins and Betaxanthins in Cultivated Varieties of *Beta vulgaris* L. Compared to Weed Beets

**DOI:** 10.3390/molecules25225395

**Published:** 2020-11-18

**Authors:** Milan Skalicky, Jan Kubes, Hajihashemi Shokoofeh, Md. Tahjib-Ul-Arif, Pavla Vachova, Vaclav Hejnak

**Affiliations:** 1 Department of Botany and Plant Physiology, Faculty of Agrobiology, Food and Natural Resources, Czech University of Life Sciences Prague, 16500 Prague, Czech Republic; kubes@af.czu.cz (J.K.); vachovap@af.czu.cz (P.V.); hejnak@af.czu.cz (V.H.); 2 Plant Biology Department, Faculty of Science, Behbahan Khatam Alanbia University of Technology, Khuzestan 47189-63616, Iran; hajihashemi@bkatu.ac.ir; 3 Department of Biochemistry and Molecular Biology, Faculty of Agriculture, Bangladesh Agricultural University, Mymensingh 2202, Bangladesh; tahjib@bau.edu.bd

**Keywords:** beetroot, beetcrops, weedbeet, betalains, spectrophotometry, storage

## Abstract

There are 11 different varieties of *Beta vulgaris* L. that are used in the food industry, including sugar beets, beetroots, Swiss chard, and fodder beets. The typical red coloration of their tissues is caused by the indole-derived glycosides known as betalains that were analyzed in hypocotyl extracts by UV/Vis spectrophotometry to determine the content of betacyanins (betanin) and of betaxanthins (vulgaxanthin I) as constituents of the total betalain content. Fields of beet crops use to be also infested by wild beets, hybrids related to *B. vulgaris* subsp. *maritima* or *B. macrocarpa* Guss., which significantly decrease the quality and quantity of sugar beet yield; additionally, these plants produce betalains at an early stage. All tested *B. vulgaris* varieties could be distinguished from weed beets according to betacyanins, betaxanthins or total betalain content. The highest values of betacyanins were found in beetroots ‘Monorubra’ (9.69 mg/100 mL) and ‘Libero’ (8.42 mg/100 mL). Other beet varieties contained less betacyanins: Sugar beet ‘Labonita’ 0.11 mg/100 mL; Swiss chard ‘Lucullus,’ 0.09 mg/100 mL; fodder beet ‘Monro’ 0.15 mg/100 mL. In contrast with weed beets and beetroots, these varieties have a ratio of betacyanins to betaxanthins under 1.0, but the betaxanthin content was higher in beetcrops than in wild beet and can be used as an alternative to non-red varieties. Stability tests of selected varieties showed that storage at 22 °C for 6 h, or at 7 °C for 24 h, did not significantly reduce the betalain content in the samples.

## 1. Introduction

The EU is the world’s leading producer of sugar beets, with a cultivation area of 1.74 million hectares (2018)—approximately 50% of the global production. The Czech Republic has a long history of growing sugar beets and, upon admission to the EU, has contributed 4.4% of the EU production. The cultivation area was 157 thousand hectares in 1980, 50 thousand hectares in 2008, and 65 thousand hectares nowadays with a yield of 71.2 t/ha [1]. Other beet crops, such as fodder beets, Swiss chard, and beetroots, are also produced, but are of lesser importance. *Beta vulgaris* L. is grown especially for the large succulent taproot, which contains many organic and inorganic compounds depending on the cultivated variety, as well as large amounts of sucrose in the case of sugar beets. Swiss chard is the exception, because it is the leaves rather than the roots that are harvested and they have a high content of vitamins and antioxidant phenolic compounds [2].

Beside sucrose, primary metabolites, and various organic acids and vitamins, plants in the genus *Beta* synthesize secondary metabolites known as betalains. These pigmented compounds were previously mistaken for anthocyanins, another important plant substance imparting a red color to the tissues. Both types of metabolites are soluble in water and they are localized in vacuoles. However, their biosynthesis and structure are different and several authors have claimed mutual exclusivity of betalains and anthocyanins in plants [3].

Betalains are composed of two basic groups: Red betacyanins and yellow betaxanthins. Betanin from the former group is the typical *B. vulgaris* metabolite and there are about 17 different betacyanin compounds described in this plant. In contrast, only about 12 betaxanthins have been identified here, and vulgaxanthin I is the primary compound in beets [4,5]. In the food industry, certain parts of the amaranth and fruits from the *Opuntia* and *Hylocerasus* cacti are utilized as a source of betalains too, and betanin itself serves as a dye (E162) in milk products [6]. Regarding the occurrence of betalains, they are in almost whole *Caryophyllales* order and some genera of fungi [7]. The number of identified pigments from this group is increasing with better analytical methods and technologies [8] and about 180 different betacyanin pigments have already been described; as in *Bougainvillea glabra*, where 146 betacyanins were detected [9].

These secondary metabolites are derived from the polar aromatic amino acid, tyrosine, through the action of enzymes to produce dihydroxyphenylalanine (L-DOPA) and betalamic acid, the precursor compound for synthesis of both subgroups of betalains. Despite the involvement of phenylpropanoids, deamination does not occur during biosynthesis of betalamic acid. The amino group nitrogen is incorporated into the heterocyclic ring. Because of the system of conjugated double bonds, betalamic acid acts as a chromophore with a bright yellow color. Through a related biosynthetic pathway, a single carbonyl group can be added with other amino acids, amines, or *cyclo*-DOPA, from tyrosine, which affects the final coloration resulting in the synthesis of betaxanthins (amino acid adducts) or betacyanins (*cyclo*-DOPA). The conjugation with *cyclo*-DOPA shifts the absorption maximum from 480 nm (yellow) to 540 nm (purple) [10]. Betaxanthin shows hypso- or batho-chromic shifts, because conjugates of amines have lower absorption maxima (A_max_) than their amino acid analogues [11]. The presence of nitrogen in the molecule and the absence of a basic chromane core are the main structural differences between these plant metabolites and anthocyanins. Betalains can also form glycosidic bonds, such as with betanidin and its glycoside betanin; the molecules can be acylated as well [6,12].

Thanks to their structure, betalains have significant antioxidant activity that can be stronger than ascorbic acid and other molecules with similar effect [13]. This activity is dependent on the origin of pigments, because plants (genus *Beta*, *Amaranthus*, *Celosia*) from family *Amaranthaceae* synthesizes betacyanin metabolites with various structural modifications such as different position of hydroxyl groups, bonded sugar units and their acylation. Molecule of betacyanin or betaxanthin group has importance as well and the latter achieved better antioxidant results, which was also related to more hydroxyl and imino groups [14]. Nevertheless, the red constituent had better results in correlation with antioxidant activity than betaxanthins in the case of juices from red beet [15].

However, betalains are relatively sensitive to environmental factors such as heat and high or low pH and oxygen, which can cause degradation of the metabolites during the processing of plant material [16]. Still, betalains are more stable in the pH range of 3 to 7 than anthocyanins, which have an optimum pH range of 5 to 6. Outside of the optimal values, there is a shift in the absorbance spectra [17] because strongly acidic or alkaline conditions cause structural changes in the molecules of betalains [18,19].

The terms weed beet and wild beet are given to the most common hybrids between the cultured and wild forms of *B. vulgaris* L. with varying genetic compositions [20]. Populations of wild *Beta* L. species are found throughout Europe, including the Czech Republic. The first occurrence was reported in 1970 from Great Britain, where fertile annual beets were found, and they have existed as weeds in commercial sugar beet (*B. vulgaris* L. Group Altissima) fields in Europe [21] for many years, causing substantial reductions in crop size. Thus, significant losses to sugar yield and quality result if these wild plants are not removed [22]. The annual wild beet plants grow much faster and have more seed production cycles throughout the season than sugar beets making them more competitive for light and nutrients. Years of unmanaged, infested fields have increased the weed seed bank, resulting in continuous wild beet growth throughout the season [20,23]. Previous research [20,24] identified these populations as either *B. vulgaris* L. subsp. *maritima* (L.) Arcang. or *B. macrocarpa* Guss. This distinction is critical because *B. vulgaris* subsp. *maritima* will readily cross hybridize with cultivated sugar beets, while *B. macrocarpa* rarely does. In Europe, populations of *B. vulgaris* subsp. *maritima* have a wide distribution as far west as the Canary Islands and moving north along the Atlantic coast and Baltic Sea [22,24].

Weed beets have distinct physiological and morphological traits, though they may not be manifested in all plants at the same time. Phenotypically the traits include a one-year life cycle, multi-germicity, root branching in older plants and plagiotropic growth [25]. Inter-row occurrence of weed beets is common but does depend on the agriculture practices during the growing period. The goal would be to identify the weed beets early and remove them; however, if these plants are grown in rows, their determination may not be easy [26]. The red color of the hypocotyls of young weed beets can be used to identify them, or the fact that they bolt earlier than sugar beets. An effective early identification method for weed beets could, thus, contribute significantly to the detection of infestation of weed beets and their removal.

## 2. Results

### 2.1. Betalain Content in Hypocotyls of Different Beet Genotypes

The betalain content, as well as the concentration of betacyanins and betaxanthins, was determined in four different varieties of sugar beet (LSB, ESB, CSB, and MSB), fodder beets (MFB) and Swiss chard (LSC). The varieties of beetroots were evaluated separately because of the high concentration of betacyanins in their extracts (the abbreviations of tested genotypes—see Table 1).

**Figure 1:**
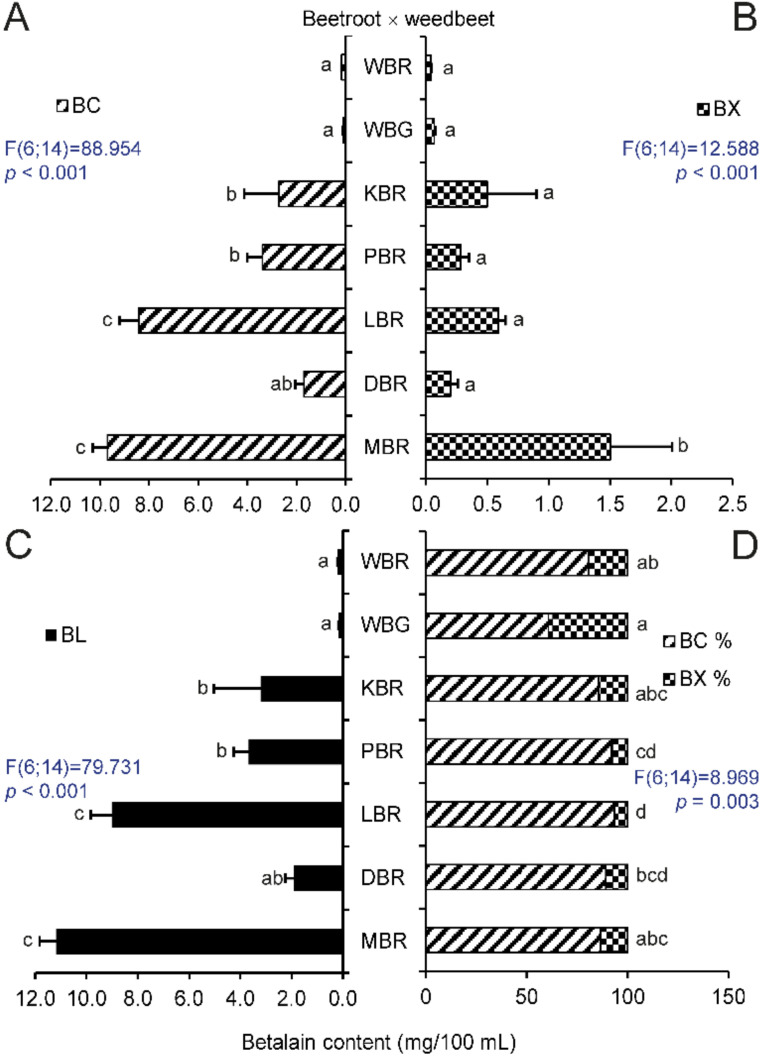
Contents of beet pigments. (**A**) BC, betacyanins, (**B**) BX, betaxanthins, and (**C**) BL, betalains, in mg/100 mL, and the BC/BX percentage ratio (**D**) in hypocotyl extracts from *Beta vulgaris* Vulgaris Group and *B. vulgaris* subsp. *maritima*. Abbreviations of specific genotypes refer to names stated in Table 1. Bars with the same letter indicate means that are not significantly different at the 0.05 probability level according to Tukey post hoc test. Horizontal lines denote standard errors.

**Figure 2:**
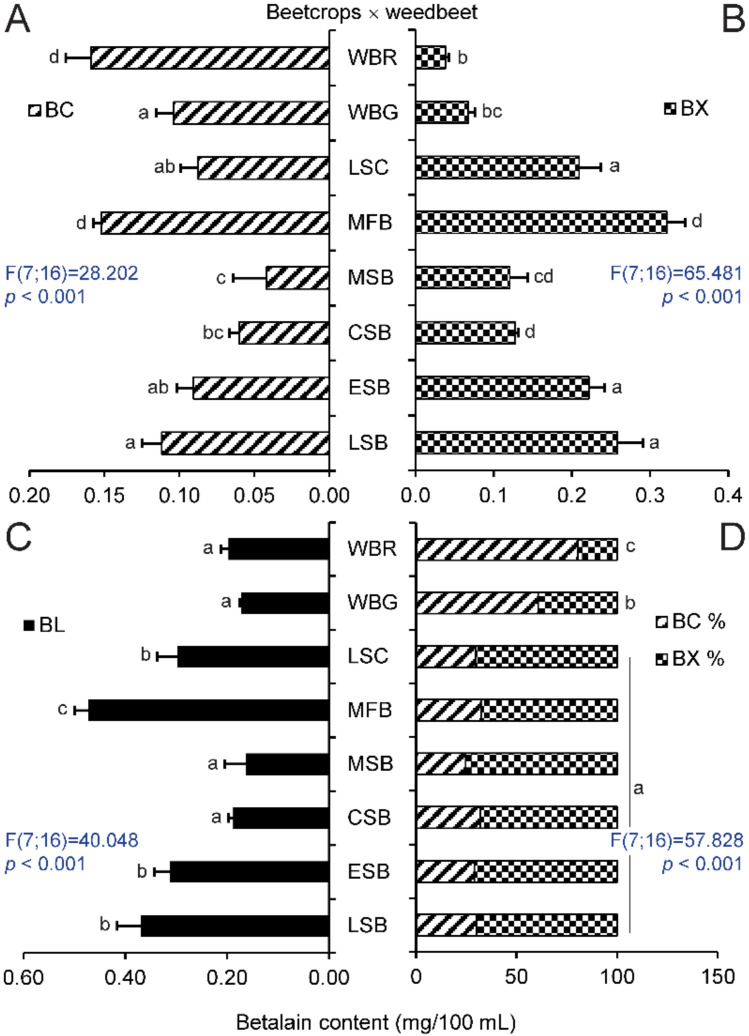
Contents of beet pigments. (**A**) BC, betacyanins, (**B**) BX, betaxanthins, and (**C**) BL, betalains, in mg/100 mL, and the BC/BX percentage ratio (**D**) in hypocotyl extracts from *Beta vulgaris* Altissima, Cicla and Rapacea Group and *B. vulgaris* subsp. *maritima*. Abbreviations of specific genotypes refer to names stated in Table 1. Bars with the same letter indicate means that are not significantly different at the 0.05 probability level according to Tukey post hoc test. Horizontal lines denote standard errors.

**Figure 3:**
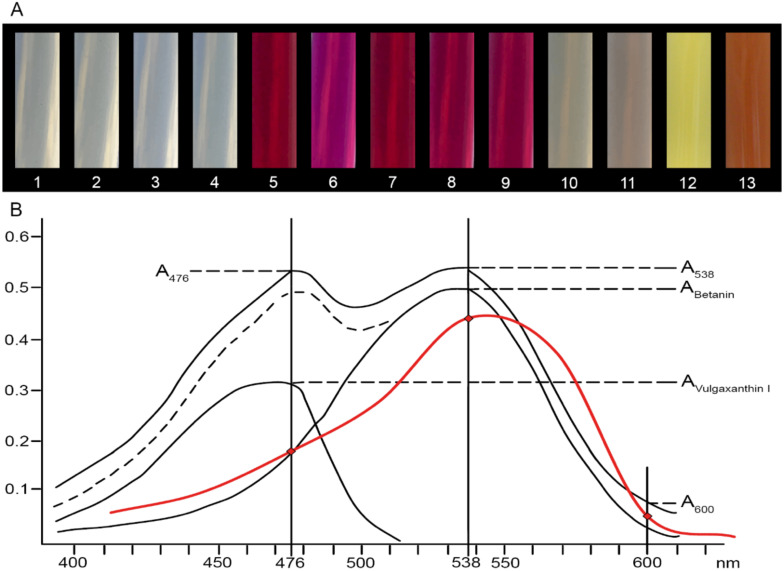
Extracts from *Beta vulgaris* hypocotyls. (**A**) Extracts of particular *B. vulgaris* L. genotypes and weed beet (1. LSB|2. ESB|3. CSB|4. MSB|5. MBR|6. DBR|7. LBR|8. PBR|9. KBR|10. LSC|11. MFB|12. WBG|13. WBR—see Table 1). (**B**) Absorbance data of beetroot extracts from *B. vulgaris* Vulgaris Group—Libero/Monorubra (red) showing the absorption spectra of betanin and vulgaxanthin (Elbe, 2001).

The level of these pigments in the prepared extracts was compared with samples from extracts from red and green hypocotyls samples of WB. The varieties of MBR and LBR had the highest concentration of betanin equivalents of all tested beets (Figure 1A). The fodder beets, MFB, contained more betacyanins than Swiss chard or sugar beets (Figure 2A), where the concentration of betacyanins was the lowest in MSB. There was strong evidence that the content of these pigments in samples of cultivated plants is distinguishable from that in extracts of WB. The only exception was fodder beet, MFB; however, this beet had more betacyanins than WB_G_. The variety, MBR, had the highest content of betaxanthins compared to other beetroots. (Figure 1B). All tested extracts of beet crops were distinguishable from WB_R_ and WB_G_ in spite of low content of betaxanthins, as they had significantly higher content of these pigments, with the exception of sugar beet, MSB (Figure 2B). As expected, MBR and LBR had the highest levels of total betalains in their hypocotyls (Figure 1C) and their extracts were darker colored than the other beets (Figure 3A). Betalains were present at greater levels in samples of MFB (Figure 2C); however, there was no significant difference in the content of these metabolites between ESB/LSC/LSB and MSB/CSB. In contrast with other beet crops, the latter varieties were indistinguishable from extracts of WB in the case of total betalains.

A recalculation of the percentage of the content of specific compounds showed that the ratio of the pigments could not be used to distinguish between individual beet crops or sugar beets. Nevertheless, all beet crops were distinguishable from WB (Figure 2D). There was also a significant difference between WB_G_ and all samples of beetroots, while no difference was seen between WB_R_ and KBR and MBR (Figure 1D).

### 2.2. Effect of Storage on Betalain Content

**Figure 4:**
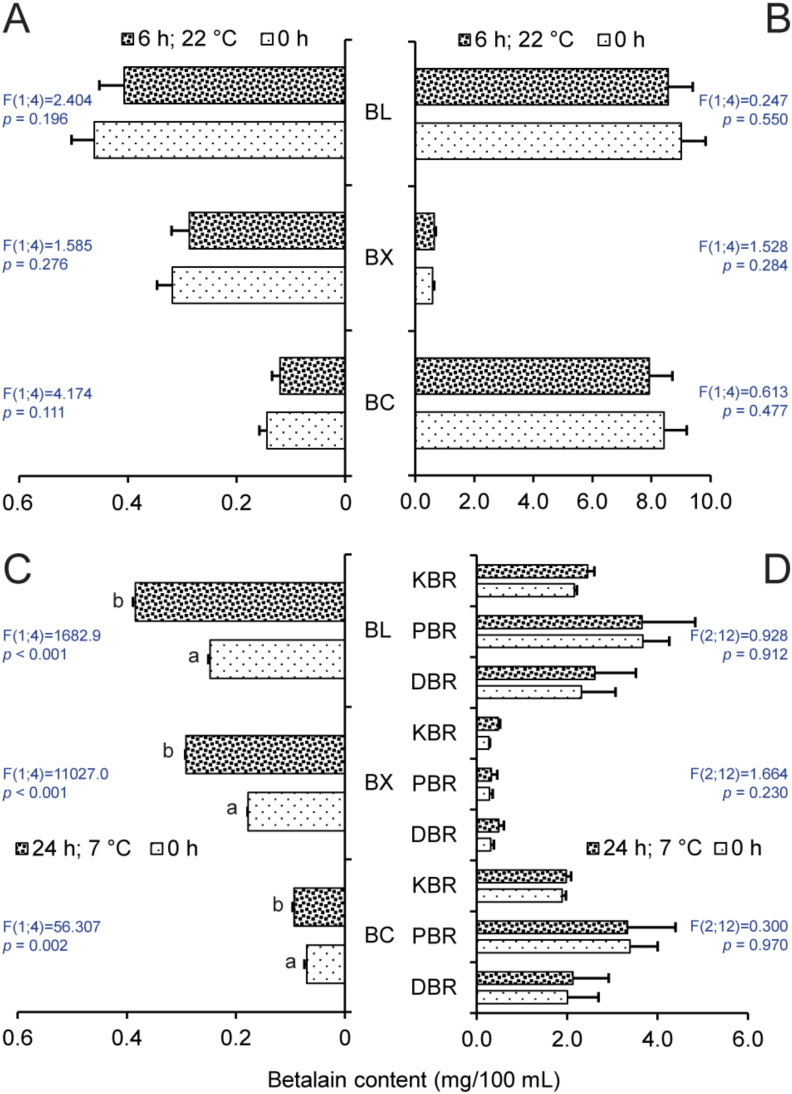
Contents of betacyanins (BC), betaxanthins (BX), and betalains (BL) in mg/100 mL in extracts from (**A**) fodder beet, MFB, (**B**) beetroot, LBR, (**C**) Swiss chard, LSC, and (**D**) and beetroots KBR, PBR, and DBR, stored at different temperatures. Abbreviations of specific genotypes refer to names stated in Table 1. Bars with the same letter indicate means that are not significantly different at the 0.05 probability level according to Tukey post hoc test. Horizontal lines denote standard errors.

Storage of MFB and LBR at 22 °C for 6 h did not significantly change the content of betalains or its individual constituents (Figure 4A,B). LBR maintained significantly higher betacyanin levels in comparison with betaxanthins, while MFB showed the opposite effect. The differences in pigmentation among the varieties were clearly visible. LSC samples stored at 7 °C contained significantly more betaxanthins and betacyanins as well (Figure 4C) after 24 h. However, higher concentrations of betaxanthins were not measured in beetroot samples, DBR, PBR, and KBR at the same conditions (Figure 4D). The prevalence of betacyanins was proven in the case of all extracts except for LSC, where betaxanthins were the primary pigment. This result further demonstrates the significant difference between beetroots and Swiss chard.

**Figure 5:**
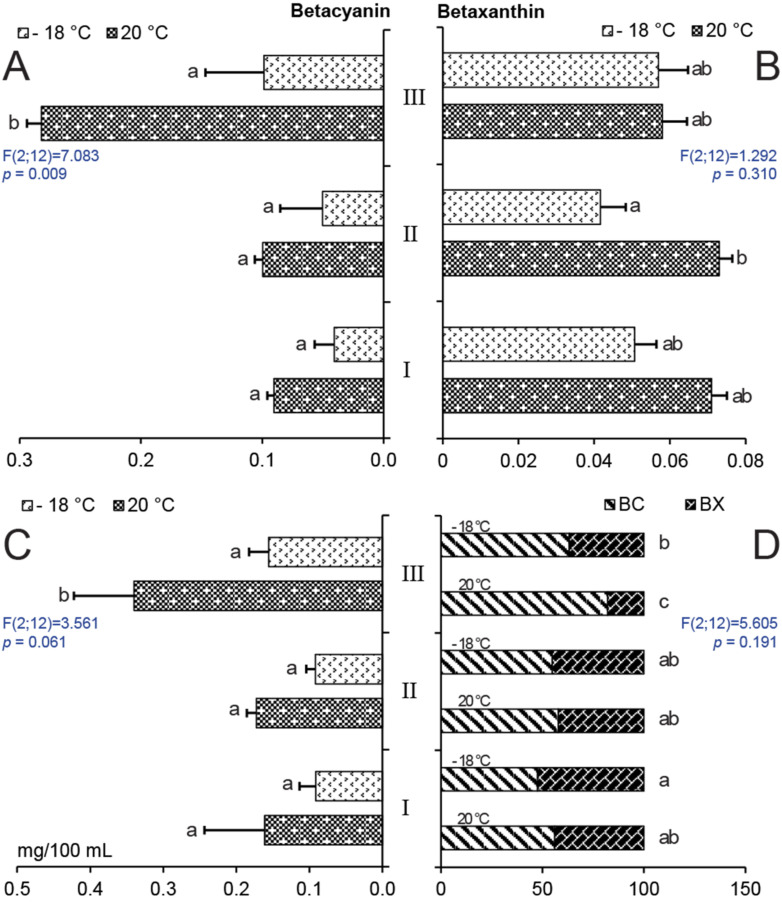
Contents of beet pigments. (**A**) BC, betacyanins, (**B**) BX, betaxanthins, and (**C**) BL, betalains, in mg/100 mL, and the BC/BX percentage ratio (**D**) in hypocotyl extracts from *Beta vulgaris* subsp. *maritima* stored at different temperatures. I—green stems, II—slightly carmine annealed stems, and III—dark crimson stems. Bars with the same letter indicate means that are not significantly different at the 0.05 probability level according to Tukey post hoc test. Horizontal lines denote standard errors.

Analysis of metabolites in WB confirmed that the red stems of sample III had the highest content of betanin equivalents (Figure 5A). Keeping samples in the freezer at −18 °C reduced the betacyanin levels by half. In one frozen sample of stem III, the content was reduced to one-third in comparison with samples held at 20 °C. The concentrations of betaxanthins were not different between individual WB samples; however, stem II had a significantly lower content of the metabolite after storage in the freezer (Figure 5B). The highest total betalain content was found in stem III and freezing decreased this by about 50% (Figure 5C). Regarding the percentage values of individual betalain components, stems I and II had similar concentrations of measured compounds and freezer storage did not change their ratio (Figure 5D). However, the content of betacyanins decreased in sample III, while a higher percentage level of betaxanthins was the result of its minimal degradation at low temperature (Figure 5B).

## 3. Discussion

In order to better identify weed beet infestation of beet crops, an improvement on the methods described in the literature that focused on the typical colorants in beetroots was used, in which total betalain content was determined in hypocotyl extracts. In agreement with previous research [15,28], higher concentrations of betacyanins and relatively low levels of betaxanthins were found in beetroot varieties LBR and MBR (Figure 1A). Other varieties had lower levels of these metabolites. Betalain synthesis is genetically fixed; however, levels can vary depending on various factors such as temperature, water content, or stage of ripening, which affect the total weight of the roots, betalain composition, and other plant characteristics [29,30]. Researchers [31] have analyzed and compared root extracts from seven different beetroot varieties. Among the different genotypes, the betalain content varied from 80 mg/100 mL to 130 mg/100 mL. The betacyanins and betaxanthins ratio was in the range 1 to 1.75. The difference in betalain concentration for individual samples of one variety was lower than that in other varieties. Our results of the analysis of hypocotyl extracts (Figure 3A) are in line with these statements, despite the lower content of betalains (MBR, 11 mg/100 mL). This apparent lack of secondary metabolites might be a result of performing the analysis on hypocotyls instead of roots and by the greater ratio of pigment subgroups. This result corresponds with one study [32], where it was stated that betalain content was dependent on the type of tissue analyzed. The concentration of these compounds in root peels of variety PBR and two other beetroot varieties was higher than in the flesh and petioles. Further research confirmed a decrease in the betacyanin content toward the center of the root, while the concentration of betaxanthins did not change in individual inner layers [5].

Other cultivated groups of *B. vulgaris* (Altissima, Cicla, and Rapacea groups) had lower total betalain content, but their samples contained more betaxanthins than betacyanins. As stated in previous research [33], beetroot hypocotyls had higher concentrations of betacyanins than Swiss chard petioles. Plant sample coloration corresponded to the content and ratio of individual pigments. However, vulgaxanthin I may not be the only betaxanthin in the sample, as shown by the metabolite screening of Swiss chard petioles [34]. The prevalent components of betacyanins and betaxanthins can be accompanied by various similar metabolites, which can affect the final color of the tissues [35]. Apart from DBR, where the ratio of pigments should be rather used, comparing cultural varieties with WB_R_ showed that betacyanins content in plant hypocotyls is a suitable indicator for weed beet identification. An alternative method utilizes WB_G_, where betaxanthin concentration can also distinguish these plants from non-red varieties such as sugar beets, fodder beets, and Swiss chard.

As stated before, these particular pigments can be determined by more sensitive methods such as high performance liquid chromatography combined with photodiode array detection or mass spectrometry. Nevertheless, according to [13] spectrophotometry represents the most straightforward method for determination of betalains content. It is with agreement that these methods related to secondary metabolites in agriculture practice should not be time and cost-consuming, but they should still carry representative information [36,37]. The application of HPLC-MS^n^ and UV-Vis spectrometry have been investigated [32], where the latter method determined betacyanins, betaxanthins, and total content of betalains in different beetroots.

Regarding the stability of the beet product (root, juice, dye), an understanding of the factors responsible for degradation is essential for maximizing the stability of betacyanins and betaxanthins in food [38], because betalains are relatively unstable pigments [39,40]. The quantity and quality of betalains in a harvested crop can be affected by the method of processing and by the subsequent storage conditions. The culinary treatment of beetroot roots, such as boiling or roasting, also has an effect on the pigment content; however, the antioxidant activity was found to be increased in this research [41]. Another study [42] described the different conditions affecting betalain stability. The stability of these metabolites is a function of their structure, and various studies confirmed the lower lability of betacyanins compared to betaxanthins. The stability of these molecules was further increased by glycosylation. Degradation of betalains in beetroots with a subsequent change of color was mainly a result of pH values that were out of the optimal range [43] or exposure to high temperatures where first order kinetic changes are involved [44,45]. These natural dyes are also sensitive to water content, light, oxygen, and the presence of certain metals [6,12].

**Figure 6:**
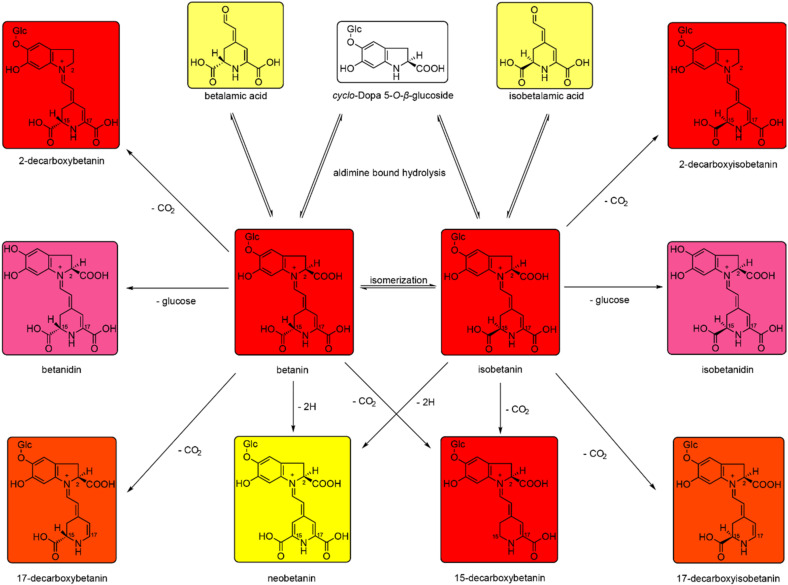
Chemical structure of betanin, isobetanin, their precursors, and degradation products. Adapted according to [46].

Certain compounds, such as isomers of betacyanins, can be formed naturally because of external conditions or their production can be supported by artificial treatment [46]. Regarding betanin, its epimer isobetanin differs in chiral center on C_15_, and has also different properties such as a prolonged retention time, but the epimer retained the same color [18]. As stated [46], the isomerization process can differ depending on the species or the betacyanin type. The loss of conjugated sugar was attributed to the activity of β-glucosidases or the effect of high temperature or low pH that led to the formation of labile aglycones with different λ_max_. Other conditions, such as pH > 6, can cause hydrolysis of the aldimine bond of betanin with production of other molecules [18]. These compounds have a different color (or no color), as is shown in Figure 6, and these changes affect the final color of the solution. Hydrolysis can also result in a loss of acyl groups in the metabolites, and the carboxyl groups of betacyanins and betalains are also susceptible to degradation [47]. However, the loss of one carboxyl group did not affect the betanidin chromophore, and the resulting molecule was even more stable [19].

Selected representatives of the above-mentioned varieties were tested in order to determine the decrease in betanin and vulgaxanthin I equivalents under different conditions. The analysis of MFB and LBR extracts showed that brief storage at room temperature, when the samples were not exposed to elevated temperature and were away from light, did not change the content of betalains or their ratios (Figure 4C). However, one study [19] stated that several hours’ exposure of samples to higher temperature had a negative effect on the stability of these metabolites in beetroots. Betanin isomerized to isobetanin in the solution and vulgaxanthin I was also degraded. Elevated temperature caused the production of neobetanin because of oxidation and the formation of a double bond between C_14_ and C_15_. Similar to betanin, this compound produces a yellow color and is present in various plant species [12]. Storage at lower temperatures [48] or protection from light [49] were presented as protective factors for betalains against degradation. While the storage of beetroots in the dark at 7 °C for 24 h had no positive or negative effect on their betalain concentration, LSC (Figure 4C) appeared to have higher amounts of betacyanin and betaxanthins. It subsequently manifested in a significantly higher content of betalains. A possible explanation of this is a slight turbidity of the solution because of the decomposition of various compounds in the extract that increased the spectrophotometric readings.

However, betalains can be also regenerated. One factor in the pigment regeneration is the presence of molecules with amine groups, which form a bond with betalamic acid. In the presence of glutamine, vulgaxanthin I can be produced instead of betanin, which alters the color of the solution [46]. Regarding protection of the betalains, positive effects was described for ascorbic acid that protected betalains during the microwave-assisted extraction [50].

As mentioned above, *B. vulgaris* is not the only plant containing betalains and many papers focused on other utilizable species. This can be a case of extracts from *Celosia argentea*, where the betaxanthins were more stable in the form of a lyophilized powder [39].

In our experiments, storage at −18 °C for two weeks caused noticeable degradation of colored compounds in solutions, and it could be hypothesized that a shorter storage time might still not have a significant effect on the content of measured metabolites (Figure 4C). Study [51] reported on the effects of different drying conditions and storage on betacyanin content in *Amaranthus* samples. The least degradation was found in samples subjected to freeze drying and storage at −18 °C, while the greatest loss was caused by drying in air and sun. However, the storage of WB stems at −18 °C caused a decrease of betacyanins concentration in sample III and betaxanthins concentration in sample II (Figure 5A,B), which resulted in a lower total betalain content (Figure 5C).

It is possible that other factors were involved in the degradation seen here. The samples with low water content were more stable [39], in comparison with our samples of WB stored in aqueous solution. *Hylocereus polyrhizus* extracts showed low degradation in the dark at 4 °C, while exposure to light and storage at room temperature had a negative effect on the amount of colored substances and samples exposed to light that were not stabilized by ascorbic acid [52]. Betacyanins from this plant used in yogurt were also more stable than the dye, E162, and showed a certain degree of regeneration during storage at 4 °C [53]. Nevertheless, the properties of the packaging material, in which plant extract or tissue is stored, can take effect on the content of the pigments with respect to oxygen transmission, despite the storage at cold temperatures [54].

Other factors, which can take a negative effect on the pigment content of beet dyes, are enzymes, such as *β*-glucosidase [55], if they are not inhibited by heat treatments such as blanching [6]. The application of high-pressure carbon dioxide (7.5 MPa) at 55 °C effectively suppressed enzyme activity in beetroot extracts [56]. The effectiveness of this method depended on the amount of betanin in comparison with classic heat treatment. High-hydrostatic pressure (650 MPa) was also tested as an alternative to blanching. The inhibitory effect on enzymes was less, but the concentration of betalains was higher with the shorter processing time [57]. In contrast, high pressure treatment of stalks from beetroot ‘Detroit Dark Red’ did not enhance betanin stability, while short heating reduced its degradation during four days of storage in plants with higher content of this metabolite, and also decreased peroxidase activity [58].

## 4. Materials and Methods

### 4.1. Plant Materials (Beta Varieties and Weed Beets)

Seeds of 11 varieties of beet crops were purchased from various seed companies in the Czech Republic. The varieties were planted in a randomized block design with three replicates each at the experimental field (49°7886111′ N, 15°3983333′ E) of the Czech University of Life Sciences Prague, Czech Republic (Table 1).

The plantings were done in two-meter-long rows with row-to-row and plant-to-plant distances of 0.45 and 0.20 m, respectively. Seeds were manually planted at a depth of 0.5 cm from 18 to 20 May 2015. Each variety was represented by four experimental rows and two control rows, grown as the border rows in each replicate in order to minimize the competition for nutrients. The spacing between the sugar beet rows was 0.45 m and all crops received uniform nutrition, irrigation, and protection. In the rosette growth phase (leaves covering 20% of the ground), beet hypocotyls were randomly selected for chemical analysis of their betalain content. Due to the instability of betalains, the samples were cleaned from soil, mixed, divided into equal groups, and immediately processed for analysis. Each experiment was repeated three times and included 10 crop beets per replicate. The weed beets (wild beets) were randomly collected from a commercial field located at Predni Kopanina (50°121720′ N, 14°292173′ E), on the northwest border of Prague, twice during 2015 in the same phase as crop beets (average temperature and precipitation during vegetation period, see Table 2). The weed beets were immediately transported to the Plant Anatomy and Morphology Laboratory in conditions protected against heat and light. The weed beet parts used in the experiment were graded based on the color: green (I), slightly carmine annealed (II), and dark crimson (III) (Figure 5). At the first time point, extracts were prepared according same procedure and then freeze-preserved for 14 days at −18 °C. On the second date, samples were processed into extracts and used for analysis immediately. It was, therefore, possible to compare the content of the unstable betalains at the two time points. The experiment used a completely randomized block design and a two-factor factorial design. Each treatment was repeated three times and included 10 weed beets per replicate.

### 4.2. Extraction and Analysis of Betalains

Determination of betalain content was performed on 50 g samples, which consisted of the hypocotyl and 2 cm of rosette. The plant material was disintegrated using an immersion blender (1015 90000 Spesso; ETA) in 150 mL of distilled water for 1 min. Subsequently, diatomaceous earth (Celite S; Sigma-Aldrich, Schnelldorf, Germany) was added to the suspensions and the mixture was filtered through Whatman No. 1 filter paper at low pressure. The filtrate was washed with distilled water until the liquid passing the filter was clear. The merged and measured volumes of water extracts. The water extracts were centrifuged at lab temperature by 10,000× *g* on MPW-251 centrifuge (MPW Med. Instruments, Warsaw, Poland) for 15 min and supernatants were filtered through a 0.45 µm membrane filter. The final solutions were analyzed immediately (Figure 1 and Figure 2) or stored at 22 °C, 7 °C, or −18 °C according to the test conditions (Figure 4 and Figure 5). Spectrophotometric analysis of extracts, prepared from different varieties as adapted from [59], was performed using a HELIOS γ spectrophotometer (ThermoFisher Scientific, Waltham, MA, USA). Samples with too high concentrations were diluted with phosphate buffer (0.05 M, pH 6.5) to an optimal absorbance of 0.4–0.5 AU. Phosphate buffer was used as the blank. Betalain absorption was determined at three different wavelengths. Betanin has its A_max_ at 538 nm, whereas the A_max_ of vulgaxanthin I is at 476 nm. Measurements at 600 nm were used to correct for the presence of impurities. Absorbance peaks at 538 and 476 nm reflect similar structures and they can be used for analysis of betalains without isolation of specific pigments. Absorption spectra (350–750 nm in 50 nm steps) were measured for LBR beetroot varieties only (Figure 3B). The absorption of betanin constituent (A_Betanin_) was determined after subtracting A_600_ (Equation (1)). The calculation for absorbance of impurities is presented in Equation (2). Betanin also absorbs light at 476 nm, and this effect is included in Equation (3) for the determination of vulgaxanthin absorption (A_Vulgaxanthin I_).

(1) $$A_{\mathrm{Betanin}}=\;1.095\times \left({\;A}_{538}-A_{600}\right)$$

(2) $$A_{\mathrm{Impurities}}=A_{538}-{\;A}_{\mathrm{Betanin}}$$

(3) $$A_{\mathrm{Vulgaxanthin}\;I}=\;\frac{A_{476}-{\;A}_{\mathrm{Impurities}}-{\;A}_{\mathrm{Betanin}}\;}{3.1}$$

Absorption of betanin at 476 nm is not constant, and changes according to the concentration; therefore, the ratio A_538_/A_476_ was utilized for the calculations and its value was 3.1. Vulgaxanthin I does not absorb light at 538 nm [59]. The concentrations of betacyanins and betaxanthins were determined using absorbance A^1%^ and the corresponding dilution factor. A^1%^ is the extinction coefficient of a 1% solution ($A_{\mathrm{Betanin}}^{1\%}$ = 1120, $A_{\mathrm{Vulgaxanthin}\;I}^{1\%}$ = 750), converted to units of mg per 100 mL with regard to the volumes of the tested extracts. The total content of betalains was calculated as the sum of betacyanins and betaxanthins (Equation (4)):

(4) $$c_{\mathrm{Betanin}}=\frac{25\;\times {\;A}_{\mathrm{Betanin}}}{1120}$$

$$c_{\mathrm{Vulgaxanthin}\;I}=\frac{25\;\times {\;A}_{\mathrm{Vulgaxanthin}\;I}}{750}$$

There are other pigments with structures similar to betanin and vulgaxanthin I, but these two metabolites represent 95% of the betalain content. According to [59], the other pigments have similar absorbance maxima, but their contribution is negligible.

### 4.3. Statistical Analysis

A mixed-model procedure, with a repeated statement for each parameter, was used to analyze the data set. Data from each variety and weed beets were analyzed using a Factorial ANOVA model with a post-hoc test of differences among combinations of experimental factors using the Tukey HSD method. All statistical tests presented in this study were performed using a Statistica 13.5 (StatSoft Inc., Tulsa, OK, USA) software package.

## 5. Conclusions

In conclusion, simple spectrophotometric measurements and calculations of the total betalain content and the percentages of specific components, such as betacyanins and betaxanthins, in samples of 11 cultivated and one wild variety of *B. vulgaris* showed that these parameters were suitable for preliminary variety verification. This method can also be utilized for the identification of weed beet infestation in beet fields. Short-term storage at normal and lower temperatures generally did not change the contents of these compounds, and samples can be stored for a certain time before analysis without misrepresentation. Further qualitative and quantitative analysis of specific pigment molecules from *B. vulgaris* L. genotypes would bring more detailed information regarding crop and wild beets.

## Figures and Tables

**Table 1:** Experimental beet crops and weed beets used.

Abbr.	Taxon *Beta vulgaris* L.	Variety Name	Crop	Variety Attributes
LSB	Altissima Group	Labonita	Sugar beet	A2011 |2|D| m
ESB	Altissima Group	Esperanza	Sugar beet	A2004 |2|D| m
CSB	Altissima Group	Caruso	Sugar beet	A2005 |2|D| m
MSB	Altissima Group	Merak	Sugar beet	A2003 |2|D| m
MBR	Vulgaris Group	Monorubra	Beetroot	A1987
DBR	Vulgaris Group	Detroit Dark Red 2	Beetroot	A1997
LBR	Vulgaris Group	Libero	Beetroot	A1994
PBR	Vulgaris Group	Pablo	Beetroot	A1994 | hybrid
KBR	Vulgaris Group	Kahira	Beetroot	A2003
LSC	Cicla Group	Lucullus	Swiss chard	A1973
MFB	Rapacea Group	Monro	Fodder beet	A1994 |4|-|m
WB *	subsp. *maritima* **	-	Weed beet	hybridizes withcultivated beets

* WB_G_—weed beets green parts | WB_R_—weed beets red parts ** or *Beta macrocarpa* Guss. |Abbr., abbreviation; A, admission in national list status; 2, diploid; 4, tetraploid; D, double hybrid; m, monogerm (Source: European Commission [27]).

**Table 2:** Average monthly temperature levels and precipitation (Year 2015) at the experimental field (‘Campus Czech University of Life Sciences,’ Prague, Czech Republic) and commercial field (‘Predni Kopanina,’ Prague, Czech Republic) site in °C and mm, respectively.

Month	Site	Temperature	Precipitation
April	Campus	8.3	29.7
	Commercial	8.6	28
May	Campus	13.2	33.8
	Commercial	13.7	44
June	Campus	16.3	32.9
	Commercial	16.5	58
July	Campus	20.9	68.4
	Commercial	18.5	75
August	Campus	22.4	8.6
	Commercial	18.0	68

Source: Czech Hydrometeorological Institute.
